# Development of naked Triticum durum × Triticum dicoccum hybrids with anthocyanin color of the grain pericarp

**DOI:** 10.18699/vjgb-26-65

**Published:** 2026-07

**Authors:** E.I. Gordeeva, A.Yu. Novoselskaya-Dragovich, O.Yu. Shoeva, E.K. Khlestkina

**Affiliations:** Institute of Cytology and Genetics of the Siberian Branch of the Russian Academy of Sciences, Novosibirsk, Russia; N.I. Vavilov Institute of General Genetics Russian Academy of Sciences, Moscow, Russia; Institute of Cytology and Genetics of the Siberian Branch of the Russian Academy of Sciences, Novosibirsk, Russia; Institute of Cytology and Genetics of the Siberian Branch of the Russian Academy of Sciences, Novosibirsk, Russia Federal Research Center the N.I. Vavilov All-Russian Institute of Plant Genetic Resources (VIR), St. Petersburg, Russia

**Keywords:** naked emmer, purple grain, anthocyanins, gliadins, marker assisted breeding, PCR markers, голозерная полба, фиолетовая окраска зерна, антоцианы, глиадины, маркер-контролируемая селекция, ПЦР-маркеры

## Abstract

The production of emmer hybrids with a high content of anthocyanins in the grains for the production of functional foods is a promising breeding direction. Phenotyping and preliminary assessment of the inheritance of gliadin-coding genes were performed for the most promising purple-grained emmer hybrids obtained previously after a complex three-stage crossing of purple-grained durum wheat (T. durum Desf.) with two different forms of spring emmers (T. dicoccum Schrank): the hybrid naked-grained variety Gremme and the red-grained awnless mutant line k25516. Genotyping hybrids for the storage protein genes in wheat grain, gliadins (Gli), enabled the selection of a purple-grained line that fully inherited gliadin-coding genes from emmer wheat k-25516, and a line inheriting these genes from durum wheat and emmer wheat k-25516. To improve the breeding material, backcrossing of three phenotypically and qualitatively different purple-grained hybrid lines with the parental variety Gremme, which demonstrated the highest yield, was conducted. During the Pp (Purple pericarp) genes selection of the plants in F2–3 progenies, the use of microsatellite markers located close to Pp genes did not demonstrate reliable linkage to the target genes. The intragenic polymorphic PCR markers made it possible to accurately select plants carrying dominant alleles of two complementarily interacting genes, Pp-B1 and Pp3 in F2–4. Based on the ease of grain threshing, the plants were selected in F4. Thus, over two years, using small areas of the greenhouse and marker-controlled selection, a collection consisting of 25 naked and semi-naked spring purple-grained lines of wheat-emmer hybrids, constant in anthocyanin coloration and differing in gliadin-coding genes and other quality traits, was obtained.

## Abstract (ru)

Одно из перспективных направлений селекции – получение гибридов полбы с повышенным содержанием антоцианов в зерновке для производства функциональных продуктов питания. Проведено фенотипирование и дана предварительная оценка по наследованию глиадинкодирующих генов наиболее перспективных фиолетовозерных полбяных гибридов, полученных нами ранее после сложного трехступенчатого скрещивания фиолетовозерной твердой пшеницы (T. durum Desf.) c двумя разными формами яровой полбы (Т. dicoccum Schrank): гибридным голозерным сортом Греммэ и безостой краснозерной мутантной линией к-25516 (коллекции ВИР). Генотипирование гибридов по генам (Gli) запасных белков зерновки пшеницы – глиадинам – позволило отобрать фиолетовозерную линию, целиком наследующую глиадинкодирующие гены от полбы двузернянки к-25516, и линию с наследованием этих генов от твердой пшеницы и полбы двузернянки к-25516. Для улучшения селекционного материала было проведено возвратное скрещивание фиолетовозерных пшенично-полбяных гибридов, содержащих доминантные аллели Pp-B1 и Pp3, с показавшим наилучшую урожайность белозерным голозерным сортом Греммэ. Произведен маркер-контролируемый отбор растений поколения F2–4 по генам Pp (Purple pericarp), регулирующим биосинтез антоцианов в перикарпе зерновки. При отборе фиолетовозерных растений в потомствах F2–3 использование микросателлитных маркеров, близко расположенных к генам антоциановой окраски, не показало надежного сцепления с целевыми генами, однако позволило проследить у гибридов сложное наследование генетического материала от разных родителей. Разработанные нами внутригенные полиморфные ПЦР-маркеры позволили точно отобрать образцы растений, несущие в ДНК одновременно доминантные аллели двух комплементарно взаимодействующих генов, Pp-В1 и Pp3. Растения отбирались по признаку легкой обмолачиваемости зерен в F4. Таким образом, за два года при использовании малых площадей тепличного комплекса при помощи маркер-контролируемой селекции в поколении F4 получена коллекция, состоящая из 25 голозерных и полуголозерных яровых фиолетовозерных линий пшенично-полбяных гибридов, константных по признаку антоциановой окраски перикарпа зерновок и различающихся по глиадинкодирующим генам.

## Introduction

The purple color of cereal grains is determined by anthocyanins, a class of flavonoid compounds. Numerous studies have shown that anthocyanins had positive effects on human health, possessing antioxidant, anti-inflammatory, hypoglycemic, and antimutagenic properties (Liu et al., 2021; Dwivedi et al., 2022; Mohammadi, 2024). Wheat grain anthocya- nins had a preventive effect against tumors and neurodegenerative diseases (Tikhonova et al., 2020, 2024; Geyik et al., 2023).

Breeding programs to develop cereals with a purple grain color as a source for the production of fortified, high-quality cereal products and pasta are attractive. One promising candidate for such nutrition is emmer (emmer wheat, Triticum dicoccum Schrank., genome BBAA, 2n = 28) – an ancient hulled wheat species, the grain of which was traditionally used in porridge. Emmer was displaced by the more productive naked-grained durum wheat due to difficulty in threshing and low yields, and as a cultivated crop, it remained in limited regions of the Volga region, Siberia, and the North Caucasus (Badaeva, 2015).

Currently, increasing interest in emmer is explained by its superior nutritional properties, genetic variability, and broad ecological plasticity (Gilev et al., 2018; Biradar et al., 2022). It is easily digestible and surpasses modern cultivated durum and bread wheat in terms of vegetable protein, unsaturated fatty acids, fiber, iron, zinc, and B vitamins, accumulating more antioxidants in the grain, making it an important component of a healthy diet (Zakharova, Tolstova, 2019; Şahin, Karakas, 2022; Cabas-Lühmann et al., 2023; Temirbekova et al., 2024). It can be used as a source for breeding wheat with enhanced nutritional qualities. A number of emmer samples are resistant to pathogens, making it possible to grow plants without the use of any chemicals or synthetic fertilizers hazardous to human health (Zverev et al., 2016; Kuznetsova et al., 2020; Biradar et al., 2022).

One of the main obstacles to the practical use of emmer wheat is the difficulty of separating the grain from its hull. In recent years, breeders have focused their efforts on developing varieties in which the grain is easily separated from the spikelet and flower hulls, making it easier to thresh. It has been proposed to classify these new emmer wheat varieties as the subspecies Triticum dicoccon (Schrank) Schübl. subsp. nudicoccon Kobyl. et Smekal. (Smekalova, Kobylyansky, 2019).

Purple-grained wheats were previously obtained through classical hybridization and selection from the tetraploid species T. aethiopicum Jakubz. from Ethiopia (BBAA genome, 2n = 28) (Zeven, 1991). The purple anthocyanin coloration trait of wheat pericarp has been well studied and is controlled by complementarily interacting anthocyanin biosynthesis regulatory genes Pp-B1 on chromosome 7B and Pp3 on chromosome 2A in tetraploid species, and by Pp-D1 on chromosome 7D and Pp3 on chromosome 2A in hexaploid species (Khlestkina et al., 2010; Tereshchenko et al., 2012). By hybridizing hexaploid wheat T. aestivum L. (BBAADD genome, 2n = 42) and performing subsequent hybrid selection using molecular markers closely linked to these genes, it is possible to easily control DNA regions carrying dominant alleles of the purple coloration genes of the grains (Gordeeva et al., 2020). In our laboratory, we have developed diagnostic intragenic DNA markers for key regulatory genes involved in anthocyanin biosynthesis in grains: Pp1-diagnostic and Pp3-diagnostic (Shoeva et al., 2022; Gordeeva et al., 2023). These markers have been successfully used to create new purple-grained wheat varieties with enhanced functional properties (Gordeeva et al., 2023).

Despite advances in hexaploid wheat breeding (Fisenko et al., 2020; Vasilova et al., 2021; Rubets et al. 2022; Shamanin et al., 2024), there are no tetraploid durum wheat varieties with high anthocyanin content in the country. Using multi-stage hybridization of two different forms of spring emmer based on tetraploid durum wheat, which accumulates anthocyanins in the pericarp of the grain, and marker-assisted selection, we created purple-grained semi-naked hybrids (Stepochkin et al., 2023). However, the main disadvantages of the new hybrids include incomplete threshing of the grain from the flower and spikelet scales, fragility of the rachis, and low yield. The emmer grains with dense scales are difficult to thresh without damaging the outer shells, which contain the main anthocyanin reserves. Moreover, this barrier dramatically reduces the breeding value of such samples. Therefore, the development of emmer lines that are naked and easier to thresh without damaging the integrity of the embryo and outer grain shell, allowing for the preservation of the beneficial properties of emmer with anthocyanin pericarp, is an urgent task.

In 2012, the Gremme spring emmer variety, which is characterized by its nakedness and was bred in Tatarstan, was included in the State Register (http://reestr.gossort. com/reestr/sort/9052467). The variety was obtained from an interspecific cross of emmer T. dicoccum Schrank. Belka variety with durum wheat T. durum Desf. Svetlana variety, followed by backcrosses on emmer (Temirbekova et al., 2020), and can serve as the starting material for the creation of naked emmer hybrids.The aim of this study was to develop new, higheryielding, naked wheat-emmer hybrids with increased anthocyanin content in the grain pericarp. For this purpose the following was performed: 1) a selective assessment of the gluten protein content of previously obtained wheat-emmer hybrids and their parents; 2) crossing of selected promising samples with the naked variety Gremme, followed by marker-assisted selection of purple-grained plants.

## Materials and methods

Plant material and crossing scheme. New lines of wheat-emmer hybrids were used in this study. The lines were obtained from complex saturating crosses of purple-grained tetraploid wheat T. durum Tri15744 with naked emmer Gremme and then with red-grained emmer T. dicoccum k-25516 at the Institute of Cytology and Genetics, Siberian Branch of the Russian Academy of Sciences (Stepochkin et al., 2023). The wheat samples used in hybridization and their origin sources are presented in Table 1. The full crossing scheme is presented in Supplementary Figure S11.

**Table 1.**
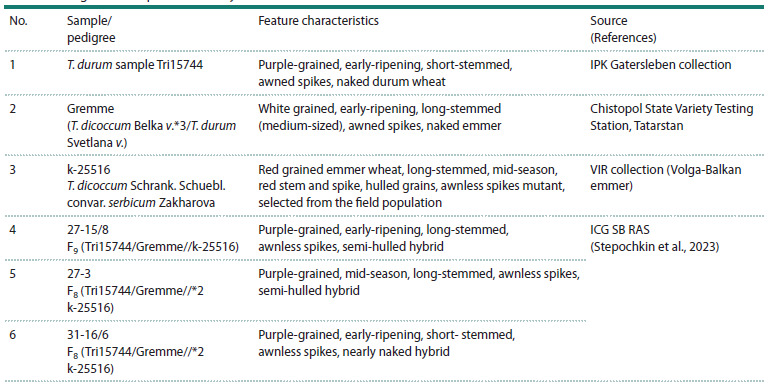
Pedigree of samples used for hybridization and their characteristics


Supplementary Materials are available in the online version of the paper: https://vavilov.elpub.ru/jour/manager/files/Suppl_Gord_Engl_30_4.pdf


To obtain completely naked, more productive plants, direct and reverse backcrosses were carried out on three contrasting samples of the hybrids we had previously obtained (No. 4, 5, and 6; Table 1), which had a rich content of anthocyanins in the grains, with naked emmer Gremme, which showed the highest productivity when grown in field conditions (Stepochkin et al., 2023). No. 4 and 6 were selected from early-ripening purple-grained samples after genotyping the genes encoding storage proteins (gliadins). Mid-season No. 5 plants showed the highest total anthocyanin content in their grains after field cultivation in 2019 (Stepochkin et al., 2023).

After backcrossing in the F2 and F3 generations, a selection of purple-grained hybrids homozygous for key genes of anthocyanin biosynthesis in the grain pericarp was carried out using phenotypic and molecular markers.

Genotyping of storage protein genes. Genotyping of hybrids during the breeding process was performed using genes encoding wheat grain storage proteins – gliadins (Gli). Gliadin-coding genes (GCGs) are characterized by multiple allelism, which allows for genotype recognition, and are localized in two of the seven chromosomes of the A genome and B genome (the Gli-A1, Gli-A2, Gli-B1, and Gli-B2 loci on chromosomes 1A, 6A, 1B, and 6B, respectively). To identify alleles of gliadin-coding loci, we used polyacrylamide gel electrophoresis (PAGE) of the gliadin from individual grains in an acidic aluminumlactate buffer (pH 3.2) (Laboratory Analysis…, 2013).

According to the catalog (Melnikova et al., 2012), the electrophoretic spectrum of each grain contains a group of polypeptides controlled by different alleles of gliadincoding loci. Three to five grains per spike were analyzed. For each electrophoresis, the homo- or heterozygous state of the alleles for each locus is provided, as well as from which parents these alleles were inherited.Cultivation, phenotyping and genotyping of hybrid material. Plant cultivation and hybridization were conducted in a hydroponic greenhouse at the shared-use center of the artificial plant cultivation laboratory and in the experimental fields of the Institute of Cytology and Genetics, Siberian Branch of the Russian Academy of Sciences (55°02ʹN, 82°56ʹE). The selection was performed in the F2 and F3 generations. The phenotypic markers such as anthocyanin coloration of vegetative and reproductive parts of plants, and DNA PCR markers for plants genotyping were used for selection.

The dark red coleoptiles coloration as a visual phenotypic marker of the dominant functional gene Pp-B1 was used (Khlestkina et al., 2008). Coleoptile coloration in 4–5-day-old seedlings soaked in Petri dishes was assessed before planting. The purple coloration of the grains as a phenotypic marker for the complementary interaction of two key dominant genes, Pp-B1 and Pp3, was used (Tereshchenko et al., 2012). Visual assessment of pericarp coloration in mature grains of the studied plants was performed after harvest.

DNA genotyping and molecular markers. DNA was extracted from young plant leaves using the method described by J. Plaschke et al. (1995). To analyze the isolated DNA of the hybrids and their parental forms, polymerase chain reaction (PCR) with intragenic markers developed for the anthocyanin biosynthesis genes Pp-1 and Pp3, as well as microsatellite markers linked to these genes, were used (Table 2; Fig. S2).

**Table 2.**
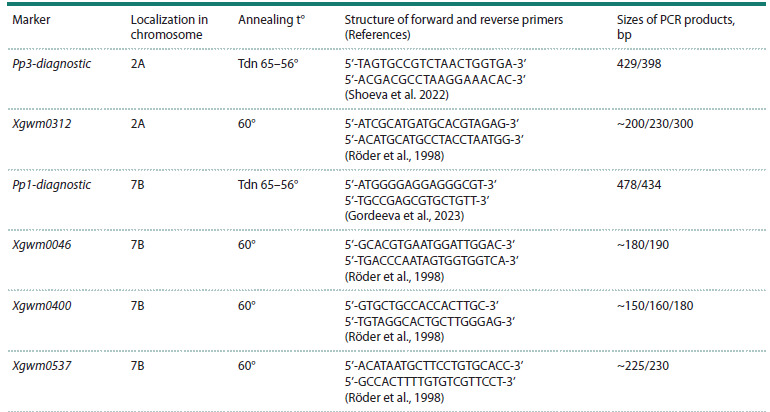
Molecular markers used in the work

To determine the substitution of chromosome regions and intervarietal inheritance of chromosomal rearrangements in hybrids, polymorphic SSR markers (simple sequence repeats) were used. These markers were selected from the group of microsatellite markers linked to the Pp3 gene on chromosome 2A – marker Xgwm0312, and to the Pp-B1 gene on chromosome 7B – markers Xgwm0046, Xgwm0400, Xgwm0537 (Gatersleben Wheat Marker) (Röder, 1998; Khlestkina et al., 2008; Gordeeva et al., 2023). The PCR conditions specified in the work of M.S. Röder et al. (1998) were followed. PCR products were separated on a 5 % HR (High resolution) agarose gel “HyAgarose™ HR Agarose” (ACTGene, Inc., Piscataway, NJ, USA).

To select wheat plants carrying dominant alleles, allele-specific diagnostic markers amplifying PCR products of different lengths together with microsatellites linked to key Pp genes were used in the study.

The intragenic allele-specific PCR marker Pp3- diagnostic, which we previously designed, allows us to clearly identify dominant and recessive alleles of the Pp3 gene in tetra- and hexaploid wheat varieties, which makes it possible to select dominant alleles of this gene in a homozygous state (Shoeva et al., 2022). Previously, six tandem repeats of 261 nucleotides in the promoter of the dominant functional allele of the Pp3 gene were identified, while only one such repeat in the recessive non-functional allele in white and red wheat varieties was found (Jiang et al., 2018).

**Fig. 1.**
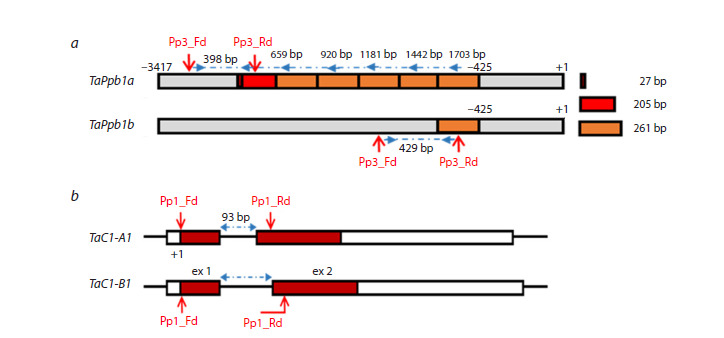
Design of intragenic PCR-markers for DNA of the Pp3 and Pp1 genes. a – scheme of differences in the structural organization of nucleotide sequences in the promoter region of functional TaPpb1a (NCBI accession number MG066455) and non-functional TaPpb1b (NCBI accession number MG066456: https://www.ncbi.nlm.nih.gov/) alleles of the Рр3 gene (Purple pericarp) based on homology to the nucleotide sequence of the Pp3 gene (NCBI accession number KJ747954). 261-nucleotide repeats of its part are shown in orange and red; b – schematic representation of differences in the structural organization of nucleotide sequences between the nonfunctional and functional alleles of Pp-A1 and Pp-B1 (syn. TaC1-A1 and TaC1-B1; (Himi, Taketa, 2015)) on chromosomes 7A and 7B, respectively. The functional allele of Pp-B1 has a longer region of a single intron between two exons, 28 base pairs longer than the corresponding region of the nonfunctional allele of Pp-A1 (blue arrows). Red arrows indicate the binding sites of the PCR primers of the Рр3-diagnostic and Pp1-diagnostic markers.

Using the PrimerQuest™Tool software, specific oligonucleotide primers were designed such that the forward primer Pp3_Fd anneals to the promoter region up to the start of the repeated regions, which differ in length and quantity between the dominant and recessive alleles, and the reverse primer Pp3_Rd anneals to the 261-nucleotide repeat. Due to the presence of truncated fragments of 27 and 205 nucleotides, the PCR products obtained using the developed primers differ in length between the dominant and recessive alleles, amounting to 398 and 429 nucleotide pairs, respectively (Fig. 1a).

The primers of the intragenic PCR marker Pp1-diagnostic flank the single intron of the Pp-B1 gene, which varies in DNA length, on both sides. This allows for the simultaneous detection of both the dominant allele of the Pp-B1 gene and the recessive non-functional alleles pp- A1 and pp-B1 (Fig. 1b). This marker was created using known nucleotide sequences of the Rc gene alleles (red coleoptile, a synonym of the Pp gene) isolated from the Chinese Spring wheat cultivar and the purple-grained isogenic line of the Novosibirskaya 67 variety (Himi, Taketa, 2015).

Diagnostic primers for the Pp1-diagnostic PCR marker were designed so that the forward primer Pp1_Fd annealed at the beginning of exon 1 for both the functional and non-functional alleles, while the reverse primer Pp1_Rd annealed 161 nucleotides from the beginning of exon 2. Due to differences in the length of the intron regions, the PCR products obtained using our primers differed in length between the non-functional and functional alleles, amounting to 472 and 434 nucleotides, respectively.

The recessive pp-B1 alleles on chromosome 7B did not differ in the length of their PCR marker products from the non-functional pp-A1 alleles. In this case, standard DNA polymerase was used to perform PCR analysis; 2 % agarose gel (LE Agarose, Lonza Rockland, Inc., Rockland, ME, USA) prepared in TAE buffer (40 mM Tris-HCl pH 8.0, 20 mM sodium acetate, 1 mM EDTA) with the addition of ethidium bromide was used to separate PCR products.

The 100 bp DNA fragment length marker (MEDIGEN Laboratory LLC) served as the standard. Electrophoresis was performed in a horizontal chamber for 1–5 hours at 7 volts/cm. UV visualization and image analysis were performed using the Molecular Imager® Gel DocTM XR+ System (Bio-Rad Laboratories, Inc., Hercules, CA, USA).

## Results

**Selection of hybrid parent plants based on gliadin-coding genes**

Initially, the parental lines were studied: T. durum Tri15744; Gremme variety, as well as T. dicoccum k-25516; the GCGs alleles were determined for each of the loci. The spectra of the parental samples differed from each other, i. e. they were controlled by different allelic variants of gliadin-coding loci, and were homogeneous, i. e. each sample had a single spectrum. During the electrophoretic analysis of the hybrid material, these samples (T. durum Tri1574, Gremme v., T. dicoccum k-25516) were used as standards necessary for the precise determination of the belonging of a block of components (allelic variant of a particular locus) to one of the three parental samples.

For plant genotyping using the GCGs method, 10 plants from each of three early-ripening purplegrained hybrid lines were selected: line 27-12, semihulled, with all normal, awnless spikes; line 31-16, nearly naked, the spikes with rudimentary awns at the top; line 31-19, semi-hulled, the spikes with an awned top (Stepochkin et al., 2023). Three to five grains from each plant were analyzed. The results of the study are presented in Table 3.

**Table 3.**
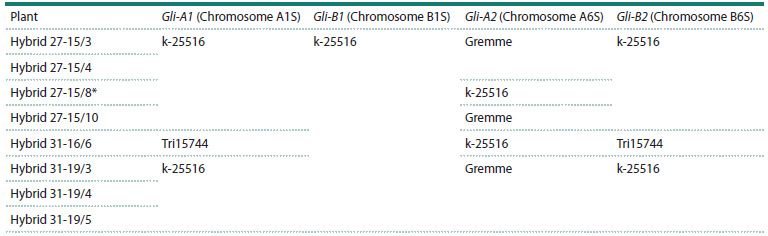
Characteristics of the emmer hybrid plants based on the inheritance of gliadins from parental forms * Inheritance of alleles of all four gliadin-coding loci from emmer T. dicoccum k-25516.

We haven’t provided genetic formulas for gliadins, as durum wheat and emmer have different nomenclatures – different catalogs of component blocks, in which the same letters denote different blocks. These catalogs haven’t yet been compiled into a unified system, thus, to ensure complete accuracy, the parent from which a particular component block/allelic variant of the gliadincoding locus was inherited is named for each locus.First, plants inheriting gliadin-coding genes from emmer T. dicoccum k-25516 were selected, and then those from naked emmer Gremme variety were selected. Only one hybrid plant, 27-15/8 (highlighted with * in Table 3, No. 4 in this paper), inherited all four gliadin-coding loci from emmer k-25516. Hybrid plant 31-16/6 (No. 6 in this paper) inherited the Gli-B1 and Gli-A2 loci from emmer k-25516, and the Gli-A1 and Gli-B2 loci from T. durum Tri1574. Plants of line 31-19 inherited 3 loci (Gli-A1, Gli-B1 and Gli-B2) from emmer k-25516 and one (Gli- A2) from naked emmer Gremme variety.

**Selection by phenotypic traits**

**Fig. 2.**
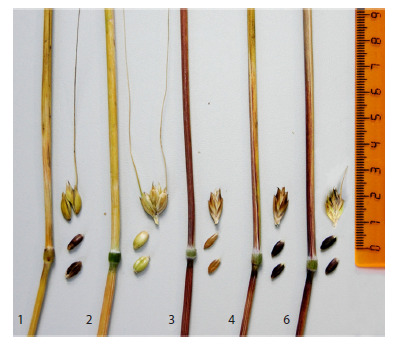
Photograph of stems, spikelets and grains of parental lines and early-ripening hybrids selected for backcrossing with the Gremme variety. 1 – purple-grained T. durum Tri15744; 2 – white-grained Gremme variety; 3 – red-grained emmer T. dicoccum k-25516; 4 – hybrid No. 27- 15/8 (pedigree: Tri15744/Gremme//*2 k-25516); 5 – hybrid No. 31-16/6 (Tri15744/Gremme//k-25516).

In addition to the purple coloration of the grain’s pericarp, plants of the hybrid lines showed intense coloration of the coleoptiles and stems (Fig. 2).

Phenotypic traits of line 27-15/8 plants (No. 4 in Figure 2) resembled emmer k-25556 (No. 3), with long, red stems at maturity and awnless red spikes. The flat

spikeletons contained two dark purple, semi-hulled, difficult-to-thresh grains.

Phenotypic traits of the dwarf line 31-16/3 plants (No. 6 in Figure 2) included rudimentary awns at the top of their compact, club-shaped, brittle ears and nearly naked grains.

Based on phenotypic traits, tall, thin-stemmed plants of line 31-19 had rudimentary awns at the top of compact, club-shaped, brittle spikes and were nearly naked. A negative trait – lodging tendency and a lower anthocyanin content in the grains (39.7 μg/g) compared to the donor T. durum Tri15744 (68.4 μg/g) (Stepochkin et al., 2023) – prevented us from further study of plants in this line.

Mid-season white-spiked plants of line 27-3 demonstrated the highest anthocyanin content in the grains (82.5μg/g) under field conditions in 2019 (Stepochkin et al., 2023) and were also selected for backcrossing with more higher yielding, naked-grained variety Gremme.

**Selection by coleoptile color**

Preliminary phenotypic (visual) assessment of coleoptile coloration in the parental plants showed that seedlings of awnless emmer k-25516, like the tetraploid donor line T. durum Tri15744, had pronounced anthocyanin coloration of coleoptiles, which corresponded to the presence of dominant alleles of the Pp-B1 gene (Fig. S3). The white-grained Gremme variety had weakly colored coleoptiles, similar to that of the hexaploid wheat variety Saratovskaya 29, associated with the presence of dominant alleles of the Pp-A1 gene, which have a weak effect on anthocyanin biosynthesis in the grains (Gordeeva et al., 2015).

After direct and reverse backcrosses of the wheatemmer hybrids with the awned, naked-grain Gremme variety, the F1 plants were self-pollinated. Sixty grains from F1 plants from each cross were soaked for germination in Petri dishes. In the F2 generation, after crossing with the Gremme variety, which carries a recessive allele of the Pp-B1 gene and, as a result, does not exhibit bright anthocyanin coloration of the coleoptiles, a visual assessment of seedling coloration was initially performed. After the F2 hybrid coleoptiles had germinated for 4–5 days, ungerminated grains and seedlings with colorless coleoptiles were discarded for planting in the ground. Only seedlings with anthocyanin-colored coleoptiles were selected for sowing.

**Molecular marker-assisted selection (MAS)**

In the tetraploid wheat T. durum Tri15744, anthocyanin biosynthesis in the pericarp is controlled by complementarily interacting genes Pp3 on chromosome 2A and Pp-A1/B1 on chromosome 7A or 7B.

**Fig. 3.**
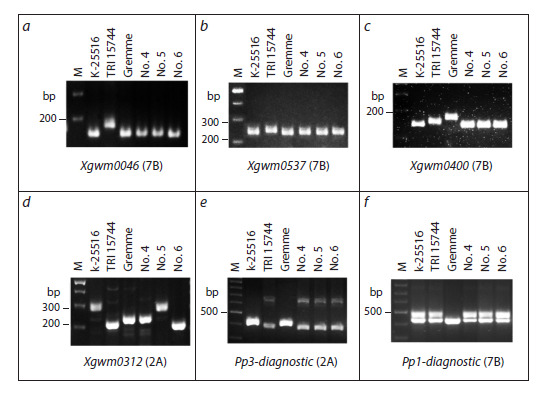
Electropherograms of PCR products obtained using microsatellite markers linked to the Pp-B1 and Pp-3 genes on chromosomes 7B and 2A, respectively, on 5 % agarose gel (a–d). Electropherograms of PCR products obtained using intragenic PCR markers to the Pp-3 and Pp-B1 genes on chromosomes 2A and 7B, respectively, on 2 % agarose gel (e–f). No. 4, 5, 6 – the DNA products of the hybrids selected for crossing with the Gremme variety; M – the length marker.

Initially, SSR markers on DNA extracted from the leaves of parental samples of purple-grained wheatemmer hybrids were tested. Microsatellite markers Xgwm0046 and Xgwm0537, located close to the target gene Pp-B1 on chromosome 7B, showed no differences between the PCR products of emmer samples k-25516 with colored coleoptiles and the Gremme variety with uncolored coleoptiles, and, consequently, no differences between the dominant and recessive alleles of Pp-B1 (Fig. 3a, b). Based on the results of PCR analysis with the Xgwm0400 polymorphic marker, all three hybrid lines (No. 4, 5, 6 in Table 1) selected for crossing with the Gremme variety inherited dominant Pp-B1 alleles only from the emmer k-25516 (Fig. 3c). For DNA genotyping of hybrids in the F2 generation, the Xgwm0400 polymorphic marker was selected. Further, for a more precise selection of purple-grained hybrids in the F3 generation, the Pp1-diagnostic intragenic PCR marker was used (Fig. 3f ).

Testing of the microsatellite marker Xgwm0312, linked to the Pp3 gene on chromosome 2A of the tetraploid wheat genome, revealed significant polymorphism and discrepancy between the PCR products of the purplegrained parental samples and the donor line of the functional Pp3 gene, T. durum Tri15744 (Fig. 3d). Therefore, selection of homozygous purple-grained hybrid samples in the F2 and F3 generations was carried out using the polymorphic intragenic PCR marker Pp3-diagnostic, which we previously developed (Fig. 3e).

After evaluating the coleoptile and grain color of F2 plants in three pairs of direct and backcross combinations, we selected five purple-grained samples from 113 plants analyzed using molecular analysis. These samples contained both dominant alleles of the Pp-B1 and Pp3 genes (Table 4). The grains of one plant were nonviable.

**Table 4.**
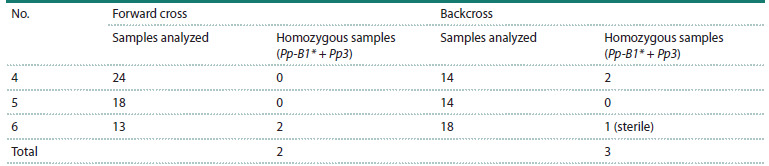
DNA analysis of F2 plants from crossing hybrids with the Gremme variety * SSR Xgwm400 served as a marker for identifying homozygous alleles of Рр-B1.

Among the 24 mature F2 plants from the cross between hybrid No. 4 and the white-grained Gremme variety, not a single dark-purple-grained plant carrying homozygous markers for both functional genes, Pp-B1 and Рр3, was found, with the PCR products corresponding only to No. 4. Among the 14 F2 plants from the backcross between the Gremme variety and emmer hybrid No. 4, two plants with dark-purple grains were found carrying homozygous markers for both functional genes, Pp-B1 and Рр3. These plants (7 and 14) (Fig. S4) had loose spikes containing five and two grains, respectively, which were sown for further generation.

Thus, using molecular analysis, we were able to select only 4 viable plants with dark purple grains, homozygous for both dominant genes, Pp-B1 and Pp3 in the F2 generation. Two plants were selected from a cross between the white-grained Gremme variety and the emmer hybrid No. 4, and two plants were from a cross between emmer hybrid No. 6 and the Gremme variety (Table 4).

For F3 planting and further analysis, dark purple grains were chosen from plants representing three groups:

1) with homozygous dominant alleles of Pp3 and heterozygous for the SSR marker Xgwm400, linked to the Pp-B1 gene; 2) with dominant alleles of Pp3, which are in a heterozygous state and homozygous for the Xgwm400 marker; 3) highly productive plants heterozygous for both the Рр3 alleles and the Xgwm400 marker.

For the control and further study, seeds of a whitegrained plant with recessive Рр3 alleles and a homozygous dominant SSR marker Xgwm400 linked to the Рр-B1 gene were selected and planted, as well as seeds of a white-grained plant with homozygous dominant Рр3 alleles and a homozygous recessive SSR marker Xgwm400. Phenotypic and molecular analysis of the plants continued in the F3 generation (Table 5).

**Table 5.**
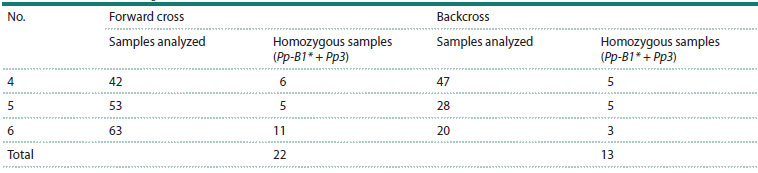
DNA analysis of F3 plants from crossing hybrids with Gremme variety * Pp1-diagnostic served as a marker for identifying homozygous alleles of Pp-B1.

In the first cross combination of hybrid No. 4 with the Gremme variety among 42 hybrids, six dark purple grained plants, homozygous for the Рр3 gene, carrying the marker of the dominant functional allele Рр-B1 were selected by using Pp3-diagnostic, Pp1-diagnostic and SSR marker Xgwm0400 (PCR products corresponded to No. 4) (Fig. S5). It was noted that one plant with dark purple grains had recessive PCR products of the SSR marker Xgwm400 linked to the Рр-В1 gene from the Gremme variety, and dominant PCR products of the intragenic marker Pp1-diagnostic (Fig. S5). This fact indicates incomplete linkage of the microsatellite marker with the target gene and the existence of a hot recombination point located between the Pp-B1 gene and the SSR marker Xgwm400.

In a backcross combination of the Gremme variety with line No. 4 in F3, based on the results of phenotypic and molecular analysis, five dark-purple-grained plants homozygous for the Рр3 gene and carrying the marker of the dominant allele Рр-B1 (PCR products corresponded to No. 4) were selected from 47 hybrid samples. In this cross combination, one white-grained plant was noted with homozygous dominant alleles Рр3 and homozygous for the SSR marker Xgwm400, linked to the Рр-B1 gene, but lacking the PCR product of the marker Pp1- diagnostic, corresponding to the dominant allele Рр-B1 (PCR products as in No. 4). This fact indicated that recombination occurred on chromosome 7B between the Pp-B1 gene and the SSR marker Xgwm400.

Thus, using molecular analysis, 35 dark purple grain plants homozygous for the Рр3 gene and containing the dominant Рр-B1 allele were selected in the F3 generation (Table 5). Grains from 29 productive plants were planted in the field, giving rise to new purple grain lines. In the F4 generation, four of the 29 lines selected exhibited a segregation for purple/white grain color (three lines inherited from family No. 5, and one line inherited from family No. 6). This indicated a heterozygous state of the dominant allele of the Рр-B1 gene in the parental samples in the F3 generation. The remaining 25 wheat-emmer hybrid lines were constant for the purple color of grains, of which seven lines had hard glumes and were difficult to thresh. The purple grains for the F4 generation giving rise to new hybrid lines were sown in the field for the selection of economic value. That is beyond the scope of this paper.

The time spent crossing the plants and selecting the appropriate hybrids was two years: three greenhouse growing seasons and two field growing seasons

## Discussion

Enriching the genetic material of tetraploid wheat with new, unique characteristics associated with the accumulation of anthocyanins in the grains and long-forgotten emmer varieties with valuable economic properties allows for expanding the industry and providing a better range of healthy food products.

**Gliadin-encoding genes**

This study examined wheat-emmer hybrids obtained previously through saturation crosses to increase the emmer genetic material, along with the naked trait.

Genotyping of the hybrids during the breeding process was performed using genes encoding grain storage proteins – gliadins (Gli). Gliadins are controlled by four major unlinked loci on chromosomes 1 and 6 of the homeologous group (loci Gli-A1, Gli-A2, Gli-B1, Gli-B2 on chromosomes 1A, 6A, 1B, 6B, respectively) (Metakovsky et al., 2019; Vinichenko, Salina, 2021). Each locus is a cluster of tightly linked genes. Genes within a single locus encode polypeptide components of different molecular weights and charges. Components controlled by a single allele are inherited in a linked manner, as a single Mendelian trait, forming a polypeptide block. The block structure is stable and does not depend on external or internal factors.

Multiple alleles have been identified for gliadin-coding loci: each locus controls over 20 alleles, allowing the theoretical identification of over one million genotypes. In fact, each variety and each line has its own unique electrophoretic spectrum, controlled by different alleles of four gliadin-coding loci (Kudryavtsev et al., 2014; Novoselskaya-Dragovich, 2015). This allows us to identify genotypes and trace the inheritance of parental alleles across generations of hybrids, determining their affiliation with specific samples at each locus.

Genotyping for gliadin-coding genes, thanks to the uniqueness of parental genotypes, makes it possible to select hybrid offspring that inherit the genotype of a specific parent, with a characteristic set of alleles at gliadin-coding loci. Analysis of the allelic composition of gliadin-coding loci in hybrid plants allowed us to determine from which parent the genes for each gliadincoding locus were inherited, i. e. to determine the degree of hybridity in lines during their creation.

To obtain a wide range of naked, purple-grained hybrids for crossing with the naked emmer Gremme variety, contrasting hybrid lines differing in their gliadin-coding gene content were selected. One of the selected lines, awnless line No. 4, inherited all four Gli loci from the awnless emmer T. dicoccum k-25516. The nearly naked, low-yielding line No. 6 inherited two loci (Gli-B1 and Gli-A2) from the awnless emmer k-25516, and two (Gli-A1 and Gli-B2) from the tetraploid wheat T. durum Tri1574.

**Crossbreeding and phenotyping**

Pyramiding multiple genes within one plant genotype is a complex process. The primary focus at this stage of breeding was on producing plants with purple-colored grains.

Anthocyanin pigmentation in wheat is regulated by two transcription factors. The R2R3-Myb-like factor, which activates anthocyanin biosynthesis in both vegetative organs (coleoptile, stem) and pericarp, is encoded by a functional allele of the Pp-B1 gene (also known as Rc-1 or Red coleoptile) (Himi, Taketa, 2015). Coleoptile coloration occurs if at least one allele of the Pp-B1 gene is dominant (Khlestkina et al., 2008). Therefore, to select plants inheriting dominant Pp-B1 alleles, visual analysis of seedlings was used before sowing. Only samples with colored coleoptiles were selected from these seedlings. The functional allele of the Pp3 gene, encoding the second regulatory factor Myc, has an endemic origin from the tetraploid wheat T. aethiopicum (Zeven, 1991). Anthocyanin biosynthesis in grains occurs only in the presence of complementarily interacting genes Pp-B1 (or Pp-D1) and Pp3 in one genotype (Tereschenko et al., 2012). While phenotyping is undoubtedly beneficial for plant breeding, the difficulty is that Pp regulatory genes are dominant and, therefore, capable of expressing coloration in seedling coleoptiles and grain pericarps in both homozygous and heterozygous states.

**Molecular markers**

Molecular PCR markers were used to select plants homozygous for both dominant alleles of the Pp3 and Pp-B1 genes, which exhibit stable purple coloration of the grains.

Testing of SSR markers on the DNA of parental samples of purple-grained wheat-emmer hybrids did not demonstrate 100 % correspondence of inheritance of the association of PCR products of microsatellites with the Pp genes. Thus, the polymorphic microsatellite marker Xgwm0312, linked to the Pp3 gene on chromosome 2A, showed a discrepancy between the PCR products of purple-grained parental accessions and the donor line of the functional Pp3 gene T. durum Tri15744 (Fig. 3d). Therefore, the polymorphic intragenic PCR marker Pp3- diagnostic, which we previously developed for hexaploid bread wheat, was successfully suited for molecular analysis during the selection of homozygous purplegrained samples of hybrids (Fig. 3e, and Fig. S4–S5). This made it possible to accurately select plant samples homozygous for the dominant alleles of the Pp3 genes in the F2–3 generations.

Testing of dark purple hybrid lines (No. 4, 5, and 6) used for backcrosses using the polymorphic SSR marker Xgwm0400 revealed the inheritance of a region of chromosome 7B, and consequently, functional alleles of the Pp-B1 gene, from the DNA of red-stemmed emmer k-25516 (Fig. 3c). This marker was used for genotyping the DNA of hybrids in the F2 generation. However, despite the proximity of this marker to the Pp-B1 gene under study, cases of incomplete linkage associated with the presence of a hotspot located between them must be considered.

In contrast to the SSR markers Xgwm0044 and Xgwm0111 on chromosome 7D, which demonstrated reliable linkage to the equivalent Pp-D1 gene locus during breeding of hexaploid purple-grained wheat (Gordeeva et al., 2020), the probability of linkage of the Xgwm0400 marker to the Pp-B1 gene locus was extremely low. This fact is consistent with the work of E. Khlestkina et al. (2008), where a short region of chromosome 7B with the target Rc-B1 (=Pp-B1) locus, which did not contain any of the nearby mapped SSR markers, was identified in inbred recombinant lines.

A hotspot breakpoint between the microsatellite marker Xgwm0312 and the Pp3 gene locus on chromosome 2A in hybrid lines inheriting introgression from the wild tetraploid species T. timopheevii Zhuk. was demonstrated by O.Y. Tereshchenko et al. (2012). This is apparently explained by a wide spectrum of genetic diversity in tetraploid wheats compared to their domesticated hexaploid relatives. Therefore, to select homozygous purple-grained hybrid samples in the F3 generation, we used the polymorphic intragenic marker Pp1-diagnostic (Fig. 3f, and Fig. S5), which we had previously designed for hexaploid bread wheat.

Since the recessive pp-B1 alleles on chromosome 7B did not differ in the length of their PCR marker products from the non-functional pp-A1 alleles, only after verifying the phenotyping of F4 generation grains were the final plant families selected that would yield new purple-grained lines homozygous simultaneously for two functional alleles of the Pp3 and Pp-B1 genes with stable, purple-colored anthocyanin-stained grains.

According to the literature, purple-grained tetraploid wheats T. durum Desf. were bred in Italy as a niche product by crossing the commercial durum wheat variety Primadur (Blondur//2587-8-6/Leeds) and T. durum Ethiopian variety T1303 (PI352395) having purple pericarp (Ficco et al., 2014, 2018). Italian scientists have also recently developed molecular allele-specific markers of the Pp-B1 and Pp-A3 (Pp3) gene loci for marker-associated breeding of purple-grained durum wheat (Esposito et al., 2024). The markers were constructed based on a comparative analysis of the primary DNA of the genotypes of purple-grained wheat T1303 and local yellow-grained durum wheat varieties Primadur and Svevo.

Similar to the results obtained in bread wheat (Jiang et al., 2018), six tandem repeats 261 bp in length were detected in the promoter region of the functional dominant allele TdPpb1 (Pp3) on chromosome 2A in purple-grained T1303, while Svevo had only one repeat. The size of the PCR products on DNA obtained from purple-grained wheat was 3,446 bp long, compared to 2,141 bp in yellow-grained varieties.

The nonfunctional TdPpm1b (Pp-B1) allele in the Primadur and Svevo varieties revealed a large insertion region of approximately 1.6 kbp, 56 bp from ATG, which disrupted the first exon. S. Esposito et al. (2024) selected primers that spanned this insertion. The PCR products obtained from the functional alleles (TdPpm1a) in purple-grained genotypes were approximately 500 bp in length, while those from the nonfunctional alleles (TdPpm1b) in local yellow-grained varieties and lines were 1,800 bp.

The intragenic allele-specific markers that we designed, Pp1-diagnostic and Pp3-diagnostic, have broader applicability and are suitable for diagnosing both hexaploid and tetraploid wheat varieties. The primers proposed in the articles by W. Jiang et al. (2018) and S. Esposito et al. (2024) amplify long PCR products that require a specialized, highly efficient polymerase for synthesis. Standard DNA polymerase is suitable for our markers.

The primers proposed in this study for the intragenic PCR marker Pp1-diagnostic, starting from the beginning of the first exon ATG, span a longer, variable-length region of the Pp-1 gene DNA. However, we did not detect long amplicons of the Pp1-diagnostic marker associated with the presence of a long insertion in the white-grained variety Gremme. It would be interesting to test the presence of this insert in cultivated varieties of durum wheat from Siberian selection.

Using a greenhouse complex during the off-season allowed us to accelerate selection and produce F4 hybrids in two years (three greenhouse and two field growing seasons). A disadvantage of greenhouse cultivation was the limited space available for sowing seeds. This resulted in a double molecular analysis and selection of the hybrid plants with the qualities we need in the F2–4 generations.

The intragenic PCR markers for the Pp-B1 and Pp3 genes that we previously designed based on the bread wheat genome demonstrated high efficiency. In small cultivation areas, the use of Pp3-diagnostic allowed for the immediate and accurate identification of homozygous genotypes of tetraploid wheat plants. The use of Pp1- diagnostic required subsequent verification of the grain color in the offspring to select samples with consistent color. These markers are a useful tool for future breeding programs.

## Conclusion

In this study, after genotyping previously developed purple-grained, difficult-to-thresh, and low-yielding emmer hybrids for their grains storage protein genes, backcrossing with the naked-grained Gremme variety and MAS selection, a set of three families of spring, naked-grained and semi-naked, purple-grained wheatemmer plants was obtained, differing in gliadin-coding genes and other qualitative traits.

Twenty-five wheat-emmer hybrid lines were finally obtained in the F4 generation. These lines differed in morphological traits and were consistent in the purple anthocyanin coloration of grain pericarp. This coloration is expressed by the presence of homozygous functional alleles of the Pp-B1 and Pp3 genes in the genotype. The presence of only one functional allele or heterozygosity leads to segregation by grains color, followed by loss of anthocyanin pigment.

## Conflict of interest

The authors declare no conflict of interest.
